# Mortality and Costs Associated with Wearable Cardioverter-defibrillators after Acute Myocardial Infarction: A Retrospective Cohort Analysis of Medicare Claims Data

**DOI:** 10.19102/icrm.2019.101007

**Published:** 2019-10-15

**Authors:** Mary Ann Clark, Steven J. Szymkiewicz, Kent Volosin

**Affiliations:** ^1^Access Strategy Partners, Braintree, MA, USA; ^2^ZOLL Medical Corporation, Pittsburgh, PA, USA

**Keywords:** Cost–benefit analysis, Medicare, mortality, myocardial infarction, wearable cardioverter-defibrillator

## Abstract

Ventricular arrhythmias are common in the early period after myocardial infarction (MI), with the highest risk occurring in the immediate postinfarct window. The wearable cardioverter-defibrillator (WCD) has been proven to have efficacy in treating sudden cardiac arrest in patients soon after MI. However, data concerning clinical and health economic outcomes of WCD usage among Medicare patients have not been evaluated. The aim of this study was therefore to investigate the clinical and health economic impacts of WCD use among Medicare patients hospitalized for MI. A 5% sample of Medicare’s Standard Analytical Files (2010–2012) was used to identify patients. Beneficiaries with an acute inpatient admission for acute MI were stratified by WCD presence and absence, respectively. Baseline clinical history, all-cause mortality, and the total cost of health-care expenditures over one year were collected. In total, 16,935 patients were included in the final analysis; of these, 89 were placed in the WCD group and 16,846 were placed in the non-WCD group. Overall, WCD patients were younger (70 versus 74 years of age; p < 0.001), more likely to be male (74.2% versus 57.4%; p = 0.002), and more likely to have congestive heart failure and/or ventricular arrhythmias prior to the indexed acute MI. At 30 days, the mortality rate in the WCD group (not reported due to volume < 11 Medicare beneficiaries) was lower in comparison with the non-WCD group (10.4%; p = 0.18). At one year, the adjusted mortality rates were 11.5% for the WCD group and 19.8% for the non-WCD group (hazard ratio: 0.46; p = 0.017). For the WCD group, the one-year incremental cost-effectiveness ratio was $12,373 per life-year gained. Among Medicare beneficiaries, WCD use after an acute MI was associated with better 30-day and one-year survival. Thus, our findings indicate that WCD use was cost-effective in the present sample of Medicare patients.

## Introduction

Ventricular arrhythmias are common in the early period following myocardial infarction (MI), with the highest risk present in the immediate postinfarct window.^1–3^ The existence of a survival benefit in post-MI patients given an implantable cardioverter-defibrillator (ICD)^4^ has been well-established. However, studies that implanted ICDs within the first months after MI failed to demonstrate an overall mortality benefit, with the presumed reduction in sudden cardiac death (SCD) being offset by an increase in the risk for nonarrhythmic death.^5,6^

The wearable cardioverter-defibrillator (WCD) is often provided to post-MI patients with reduced left ventricular ejection fraction (LVEF) until such individuals can be optimized on guidelines-directed medical therapy prior to an ICD implantation decision. A number of observational studies have reported benefits associated with WCD use in this patient population. In a study that used propensity modeling to compare clinical outcomes following coronary revascularlization with or without WCD usage, Zishiri et al. found that the majority of study participants had a history of MI and that WCD use was associated with both short- and long-term mortality reductions, with a 39% risk reduction observed during a mean follow-up period of 3.2 years.^7^ Elsewhere, Sanders et al. developed a Markov model to assess the economic benefit of using a WCD in acute MI patients. They analyzed the cost-effectiveness of various clinical scenarios and reported a result in favor of WCD deployment. Using a lifetime perspective, WCD prescription costs $11,503 more than the conventional treatment strategy but improves life expectancy by 0.261 life-years, yielding an incremental cost-effectiveness ratio (ICER) of $44,100 per life-year gained. WCD use remains cost-effective down to a per-patient sudden death risk of 1.16%.^8^

Despite the above findings, one consideration to keep in mind is that results from these studies may not be directly applicable to Medicare patients, as these individuals are older than the average WCD patient and may have a higher incidence of medical comorbidities. Singh et al., by analyzing Medicare claims data from 1992 to 2010, reported a 30-day post-MI mortality rate of 18.5%.^9^ The United States national average for 30-day mortality following MI hospitalization was 14.1%, as reported on the Centers for Medicare and Medicaid Services–affiliated website Hospital Compare (https://www.medicare.gov/hospitalcompare/search.html) for a period ending in June 2015.^10^ More recent American Heart Association data suggest that the one-year mortality rate after MI for patients aged 65 years and older was 25% to 30%.^11^

The purpose of the present study was to evaluate specifically the one-year mortality and the total cost of health care delivered to a group of risk-matched Medicare patients with and without WCD use. The second objective was to evaluate whether the use of WCD in Medicare patients following MI renders similar survival and cost-saving benefits as compared with those found in prior published reports.

## Methods

### Data sources

We utilized a 5% sample of Medicare’s 2009 through 2012 Standard Analytical Files (SAFs), which contain all parts A and B claims [ie, from physician, inpatient, outpatient, skilled nursing, home health, hospice, and durable medical equipment (DME) suppliers]. The 2010 to 2012 data were used to identify MI patients, classify them based on the presence or absence of WCD, and collect their clinical and economic outcomes for one year or until death. Incomplete claims were censored. For patients identified in 2010, 2009 data were used only to determine baseline demographic information and the presence of cardiovascular risk factors (one-year look-back period).

### Patient selection

**Figure 1:**
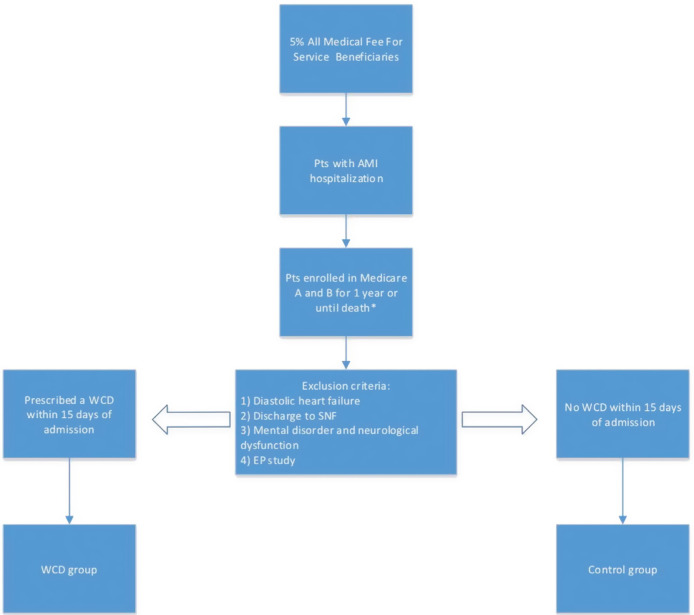
Patient selection flowchart. Each square from top to bottom represents a step during which patient records were filtered based on study design. The asterisk denotes that one-year follow-up was censored for patients with index events in 2012.

Medicare fee-for-service beneficiaries with an inpatient admission for acute MI [principal International Classification of Diseases, ninth revision (ICD-9) diagnosis code: 410.XX] and with enrollment in both Medicare parts A and B were included in this study. Patients were stratified into two groups based on the presence or absence of a WCD. The WCD group contained patients who were prescribed a WCD within 15 days of hospital discharge (Healthcare Common Procedure Coding System code: K0606) and who had a diagnosis of acute MI on their Medicare DME WCD claim. Patients with a diagnosis code for diastolic heart failure were excluded. Also excluded were patients with a claim for an electrophysiology study, those with a neurological dysfunction diagnosis, those with mental disorders that affect cognitive abilities, and/or those who were discharged directly to skilled nursing facilities (SNFs). Since some of the patients with these comorbidities may not be WCD candidates, individuals who met any of these criteria were excluded from both groups to ensure a more accurate comparison. A detailed patient selection flowchart is shown in **Figure 1**.

### Measured outcomes

The primary outcomes for this study were total all-cause mortality and total costs of health care over the one-year period after the patient’s index MI event. Index event dates were defined slightly differently for WCD patients and for those without WCDs, respectively. The index event date for non-WCD patients began with the date of hospital discharge after an acute MI, while, for WCD patients, the index event date began with the date of WCD use (which was up to 15 days after hospital discharge for acute MI).

Additional outcomes measured included health-care resource use (ie, number of claims and costs by various provider types), number of acute inpatient and SNF days, 30-day readmission rate, number of diagnostic cardiology tests, number of sessions of cardiac rehabilitation, ICD implant rate, and other clinical outcomes as identified through ICD-9 diagnosis codes. Outcomes were evaluated at 30, 90, 180, and 365 days after the index event. Costs were assessed strictly from a Medicare payment perspective for all types of services and providers and excluded any beneficiary cost sharing. All data were deidentified to protect the study participants. Institutional review board approval was not sought or deemed needed for this study since the source of data for this analysis was deidentified Medicare claims data.

### Statistical analysis and risk adjustment

Univariate analysis results are reported as means and frequencies/percentages with a t-test or Fisher’s exact test applied where appropriate. For Kaplan–Meier survival analysis, a significance level of 0.05 was used to discern differences between the groups. In accordance with standard Medicare Data Use Agreements, any data with values or calculations resulting in cell sizes of less than 11 Medicare beneficiaries are reported in this study as “NR” with a plus (+) or minus (−) sign included to indicate whether the value is above or below the value in patients with WCDs.

Multivariate analyses included propensity-score matching (PSM) to assess differences in cost and resource use between the two patient groups. Cox regression modeling was used to predict mortality in the full patient sample (and to control for the censoring of incomplete follow-up data), with hazard ratios (HRs) and 95% confidence intervals reported. Multiple covariates were assessed to adjust for baseline differences between the two groups. Starting with a combined list of variables for both the Cox regression and the PSM, variables that were not significant were removed, leaving a final list of adjusters for the two different analytic approaches. Since the analyses applied predict different outcomes (ie, Cox regression predicts mortality, while PSM predicts group membership), the lists of significant variables for the two analyses were different.

In the Cox regression model, mortality was the dependent variable, and the final independent covariates were treatment with or without WCD; age; index procedure (ie, percutaneous coronary intervention or coronary artery bypass grafting); comorbidities (eg, congestive heart failure, cardiorespiratory failure, pneumonia, renal disease, hypertension, diabetes, metastatic cancer, solid tumor, obesity, depression, coma); and hierarchical condition category variable (eg, community score, new enrollee score).

Conversely, the final matching variables used in the PSM analysis were age; index procedure; comorbidities (eg, melanomas, prostate cancer, peripheral vascular disease, aneurysms, decubitus ulcers, nephritis, vertebral fractures, drug/alcohol psychosis, respirator dependency, heart failure); and resource use (eg, chest imaging, coronary artery bypass grafting/percutaneous transluminal coronary angioplasty, other prior service, having been seen by an internist or a neurologist).

PSM was maintained at one-to-one in order to limit bias. Furthermore, in this study, PSM was a hybrid between a greedy and an optimized match. First, a greedy, one-to-one match without replacement with a caliper of 0.0001 was applied. The caliper was iteratively increased to 0.001 and, finally, to 0.01. Any remaining cohort members were excluded from the match. The propensity score was estimated using logistic regression with the dependent variable being group membership. The differences in cost were correlated with risk reduction in mortality using the ICER. The ICER was calculated as the dollar amount of the difference in cost divided by the difference in one-year mortality. The result was compared with published societal willingness-to-pay (WTP) thresholds.^12,13^

## Results

A total of 16,935 patients met the study requirements and were included in the final analysis. Among them, 89 patients were placed in the WCD group and 16,846 were placed in the control (non-WCD) group.

### Baseline clinical profile

WCD patients, with an average age of 70 years, were typically younger than those in the non-WCD group (74 years; p < 0.001). The proportion of female patients was higher in the non-WCD group (42.6% versus 25.8%; p = 0.002). The reasons for entering Medicare were comparable between the two arms. Similar rates between groups were observed for comorbidities such as atrial fibrillation, diabetes, hypertension, and hemodialysis initiation. WCD patients were more likely to have a preinfarction history of congestive heart failure (78.7%) and ventricular arrhythmias such as ventricular tachycardia (VT) (37.1%) and ventricular fibrillation (VF) (NR) prior to the indexed MI **(Table 1)**.

### Clinical outcomes

Of the 16,935 initial patients, a total of 11,465 patients had complete one-year follow-up data available and were included in the clinical outcomes assessment. Within 30 days post-MI, a greater proportion (NR) of WCD patients experienced cardiac arrest, whereas 4.8% of non-WCD patients experienced the same (p = 0.048). However, the 30-day mortality was lower (NR) in the WCD group than the 30-day mortality in the non-WCD group, which was 10.4%.

**Table 2** summarizes the one-year unadjusted clinical outcomes. While patients with and without WCDs experienced similar rates of recurrent MI (29.2% and 23.0%, respectively) and cardiac arrest (NR+ and 7.2%), syncope was more common in WCD users (NR+ versus 8.3%; p = 0.001).

Post-MI ventricular arrhythmias coded as cardiac arrest, VF, and VT were also more common in the WCD group than in the non-WCD group. At one year, prior to adjusting for baseline imbalances in covariates between the groups, WCD patients had a death rate (NR−) that was significantly lower than the 21.0% mortality rate of the non-WCD patients (p = 0.03).

**Figure 2:**
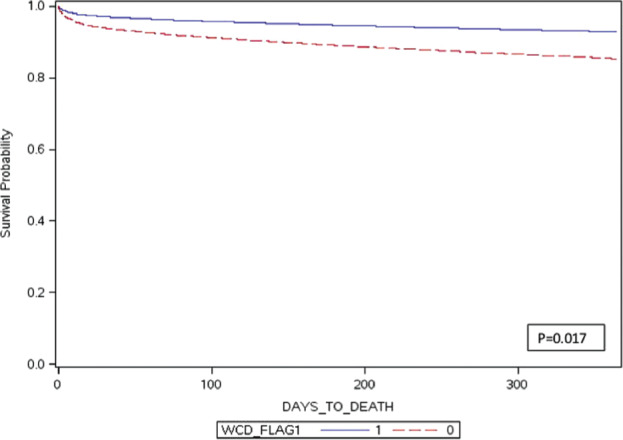
Risk-adjusted one-year mortality. The x-axis indicates survival days, while the y-axis indicates the probability of survival. Additionally, the solid line represents patients with WCDs and the dashed line represents those without WCDs.

Using Cox proportional hazard analysis, which adjusts for baseline differences in patient characteristics and controls for censored data, WCD use was still associated with a lower one-year mortality rate (HR: 0.46; p = 0.017) and was statistically significant. This calculation indicates that the WCD group had about half the chance of dying within one year following an acute MI as compared with non-WCD users. The one-year risk-adjusted death rate was 11.5% for the WCD group and 19.8% for the non-WCD group—a statistically significant finding (p = 0.0167). In the WCD group, the absolute mortality risk reduction was 8.3% and the relative risk reduction was 41.9%. The one-year survival curve is shown in **Figure 2**.

### Medical resource use

**Table 3** summarizes medical resource usage trends from PSM results. At the end of one year, WCD patients had 6.8 DME claims per person, whereas non-WCD patients had 3.6 DME claims per person (p < 0.0001). This difference was anticipated, as the WCD is reimbursed through the DME system, with each month of WCD wear represented by a separate DME claim. WCD patients also had more outpatient visits, ICD implants, and cardiac diagnostic tests but fewer hospice claims than their non-WCD counterparts. WCD and non-WCD patients both had similar numbers of cardiac rehabilitation visits, follow-up days, hospital days, and SNF days, with none of the differences being statistically significant.

**Figure 3:**
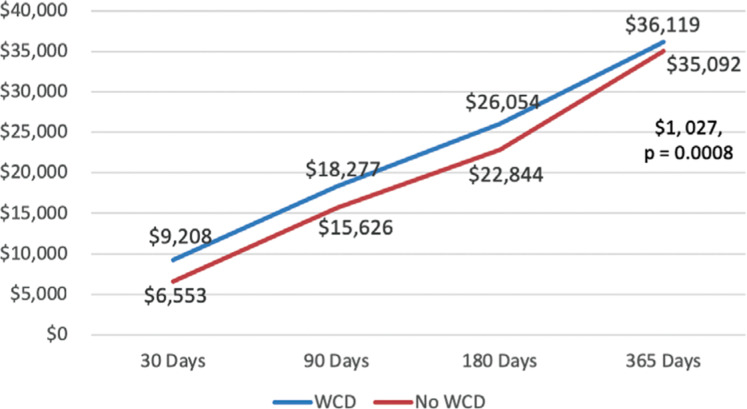
Average total follow-up cost per patient over one year as sourced from PSM analysis. The x-axis indicates the number of follow-up days since the index event, while the y-axis indicates total health-care resource utilization in United States dollars. Additionally, the green line represents patients with WCDs and the blue line represents those without WCDs.

When looking at the one-year cost to Medicare according to the PSM analysis, WCD patients spent more on DME claims ($6,652 versus $315; p < 0.0001) and outpatient services claims ($9,183 versus $2,274; p = 0.0008). Conversely, non-WCD patient spending was more for inpatient stays ($20,336 versus $12,989), physician and part B carrier services ($5,918 versus $4,909), and SNF care ($3,168 versus $413), although these differences were not statistically significant. Overall, the cost per WCD-treated patient was $36,119 annually and $1,027 more than that for a non-WCD-treated patient ($35,092). **Figure 3** illustrates the trend in WCD and non-WCD health-care costs in PSM groups of patients over a one-year follow-up period. When combined with the observed mortality reduction (19.8% – 11.5% = 8.3%), the use of a WCD costs $12,373 to save a life during the first year after MI, or an ICER of $12,373 per life-year is gained just for the first year.

## Discussion

Survival is one of the most important outcomes following acute MI. Public reporting of 30-day mortality following acute MI hospitalization has documented a slight decline in short-term mortality from 16.6% to 14.1% over time.^9,10^ The one-year mortality rate following hospitalization for acute MI in Medicare patients has been documented as 25% or higher.^11,14^ The current study demonstrated a lower mortality rate among more than 16,000 Medicare patients with acute MI (2010–2012) than previous national estimates. Although the 30-day unadjusted death rate in the WCD group was lower than the 10.35% rate in the non-WCD group, it was not significantly so. However, at one year, the risk-adjusted death rates were 11.5% for WCD patients and 19.8% for non-WCD patients, translating to a one-year relative risk reduction of about 42%.

Several population differences may have contributed to the mortality differences seen in the comparison of this study and prior reports, including age at the time of acute MI and improvements in medical care over time. This study cohort was somewhat younger than those included in previous studies (74 versus 76 years). In particular, members of the WCD group in this study were significantly younger, with an average age of 70 years. The current study cohort also had more revascularization procedures performed, which may have improved the one-year survival rate.

Of note, the WCD group demonstrated better survival than did the non-WCD group in our study. Several potential reasons may play a role in this. First, the WCD successfully prevents ventricular arrhythmic death in some patients. The risk of lethal arrhythmias is highest early after an acute MI, which occurs during guidelines-recommended waiting periods before the determination of whether or not to pursue ICD implantation for primary prevention. In the Valsartan in Acute MI Trial (VALIANT) study, the overall rate of SCD during the first month after acute MI with heart failure was 1.4% per month, increasing to 2.3% per month among those with an LVEF of 30% or less.^2^ Epstein et al. reported that 1.6% of acute MI patients using a WCD had sustained VT/VF, with a median of 16 days from the index MI to the time of first treatment (and a median of nine days from WCD prescription to first treatment).^3^ The recently published Vest Prevention of Early Sudden Death Trial (VEST) study, in an on-treatment analysis, found a reduction in sudden arrhythmic death and total mortality in patients wearing the WCD as compared with those not prescribed the WCD or who did not wear the WCD despite it being prescribed.^15^

In our study, the WCD group was clearly at a high risk for ventricular arrhythmias. At baseline, 53.9% had a diagnosis code for either VT or VF within the one year prior to the index MI event. During the one-year follow-up, 62.5% had a VT or VF event and 12.5% had cardiac arrest. Although the claims data lack clinical details, it is reasonable to expect that the WCD contributed to reducing arrhythmic mortality before the evaluation of the long-term risk of SCD was completed and the need for an ICD was determined. It should also be noted that the ICD implant rate is low in eligible Medicare patients.^16^

Second, the Medicare WCD patients may have received better follow-up care after MI. The results pertaining to medical resource use in the current study revealed that WCD patients had more cardiac diagnostic tests administered in comparison with the non-WCD group during the one-year follow-up period. The WCD patients also had more claims for physician services, outpatient visits, and cardiac rehabilitation. A recent publication by Mirro et al. reported better compliance with outpatient follow-up among WCD patients than among non-WCD patients.^17^

On average, patients in the WCD group cost $1,027 more to treat than those in the non-WCD group. A detailed analysis of the WCD group shows they spent more money in the DME category, presumably due to the costs of the WCD itself. The WCD group also had higher expenditures in the outpatient category, possibly due in part to care being shifted to an ambulatory setting from an inpatient setting. The WCD group spent half as much as the non-WCD group in the inpatient, SNF, and hospice combined components of care ($13,401 versus $25,650). When evaluating the costs together with the risk reduction, the ICER was $12,373 per life-year gained. This ratio is substantially lower (assuming no difference in quality-of-life utility measures) than the commonly accepted societal WTP thresholds, which range between $50,000 and $100,000.^12,13^

Sanders et al. evaluated the cost-effectiveness of WCD use in acute MI patients using a Markov model. Their study outcomes favored the WCD, revealing a health care system cost per life-year gained of $44,100 and a cost per quality-adjusted life-year of $60,600.^8^ Their modeled ICER was different from ours in that they chose a lifetime horizon calculation, whereas the present study incorporated a one-year follow-up determination. In addition, the Markov model that Sanders et al. employed relied on literature-based assumptions of expenditures with little real-world expenditure data. The current study provides a more detailed insight by relying exclusively on actual expenditures.

### Study limitations

The Medicare claims data do not contain detailed clinical information such as LVEF values. Thus, given that WCD users uniformly had LVEF values of 35% or less due to the Medicare coverage policy, we could only attempt to accurately select control group participants by excluding codes that are likely to relate to patients with higher ejection fractions such those with pure diastolic heart failure. Also, this study was based on a 5% sample of the Medicare population, and a different sample may result in different costs and calculated results.

Separately, the WCD users were younger and were more likely to have an arrhythmia code appear in their Medicare claims data. In our opinion, these differences likely reflect prescription biases. We chose to include all of the data available and use statistical methods to adjust for differences rather than adopt another study design such as case–control.

Medicare claims data do not contain cause of death or WCD treatment data. Without such information, the specific reasons for the differences in death rate between the two groups cannot be conclusively determined. Additionally, the Medicare SAFs did not contain any part D pharmacy claims; thus, consideration of this was excluded for both groups. Finally, because this study used the 5% SAF sample, the number of WCD patients was limited for the years included in the study and the values of several important outcomes variables could not be reported due to Medicare’s data use agreement requirements, which are designed to protect patient privacy.

## Conclusion

Using a 5% sample of Medicare’s SAFs, this study revealed that WCD use was associated with a nonsignificant trend toward a higher 30-day survival rate and a significant improvement in the one-year survival rate after acute MI. Although WCD use was correlated with higher DME costs, which was anticipated, as the WCD is reimbursed through the DME system, WCD use was also correlated with lower expenditures for other health-care categories such as inpatient stays, physician services, and SNF utilization. The purpose of this analysis was not to determine the cost-utility (cost per quality-adjusted life-year gained) of the WCD. However, the calculated ICER of using the WCD was found to be $12,373 per life-year gained. This is well below the generally accepted WTP thresholds if we assume similar quality-of-life benefits exist in patients with and without WCDs.

## Figures and Tables

**Table 1:** Baseline Patient Characteristics and Statistically Significant Risk Factors

Baseline Patient Characteristic	Patients with WCDs (n = 89)*	Patients without WCDs (n = 16,846)	p-value
Mean age	69.9 years	74.1 years	0.000
Female gender	25.8%	42.6%	0.002
Disabled	NR+	11.4%	0.338
With Medicaid	NR+	16.7%	0.978
Black	NR+	8.1%	0.885
Index event percutaneous coronary intervention	78.7%	50.7%	< 0.0001
Stress test	28.1%	18.0%	0.013
Paroxysmal ventricular tachycardia	37.1%	7.8%	< 0.0001
Tachycardia, unspecified	NR+	6.9%	0.014
Atrial flutter	NR+	3.6%	0.032
Ventricular fibrillation	NR+	3.2%	< 0.0001
Other premature beats	NR+	4.1%	0.001
Cardiorespiratory failure and shock	39.3%	26.0%	0.004
Cerebral hemorrhage	NR+	0.6%	0.033
Congestive heart failure	78.7%	45.1%	< 0.0001
Coronary atherosclerosis/other chronic ischemic heart disease	100%	87.1%	0.000
Nonpsychotic organic brain syndrome/conditions	NR+	1.7%	0.047
Other digestive and urinary neoplasms	NR+	7.5%	0.031
Other frailty condition	NR−	22.6%	0.021
Precerebral arterial occlusion and transient cerebral ischemia	23.6%	16.2%	0.059
Specified heart arrhythmias	55.1%	35.3%	< 0.0001
Unstable angina and other acute ischemic heart disease	78.7%	53.4%	< 0.0001
Valvular and rheumatic heart disease	46.1%	30.9%	0.002
Vascular disease with complications	NR+	5.4%	0.051
Viral and unspecified pneumonia, pleurisy	33.7%	20.8%	0.003

NR: not reported per Centers for Medicare and Medicaid Services guidelines, since the percentage represents fewer than 11 Medicare beneficiaries.

*Plus (+) and minus (−), respectively, indicate whether the value is above or below the value in patients without WCDs.

**Table 2:** One-year Unadjusted Clinical Outcomes

Clinical Outcome	Patients with WCDs (n = 48)*	Patients without WCDs (n = 11,417)	p-value
Death	NR−	21.0%	0.03
Acute MI	29.2%	23.0%	0.31
Ambulance	52.1%	39.1%	0.07
Cardiac arrest	NR−	7.2%	0.16
Syncope	NR+	8.3%	0.01
Paroxysmal ventricular tachycardia	52.1%	8.9%	< 0.0001
Ventricular fibrillation	NR+	2.8%	0.001
Other specified cardiac dysrhythmia	31.3%	20.1%	0.05
Cardiac dysrhythmia, unspecified	NR+	10.5%	0.02
Acute hemodialysis	NR−	25.8%	0.08

MI: myocardial infarction; NR: not reported per Centers for Medicare and Medicaid Services guidelines, since the percentage represents fewer than 11 Medicare beneficiaries.

*Plus (+) and minus (−), respectively, indicate whether the value is above or below the value in patients without WCDs.

**Table 3:** One-year Unadjusted Medical Resource Usage from PSM Analysis

Resource Used	Patients with WCDs (n = 44)	Patients without WCDs (n = 44)	p-value
DME (mean claims per patient)	6.8	3.6	< 0.0001
Physician and part B carrier (mean claims per patient)	49.2	51.9	0.63
Home health (mean claims per patient)	0.8	0.5	0.03
Hospice (mean claims per patient)	0	0.7	0.02
Inpatient (mean claims per patient)	1.0	1.0	0.30
Number of hospital days (inpatient) (mean per patient)	4.4 days	8.7 days	0.18
Outpatient (mean claims per patient)	7.2	5.7	0.05
Emergency room (mean claims per patient)	1.1	0.5	0.14
Skilled nursing (mean claims per patient)	0.1	0.4	0.19
Number of SNF days (mean per patient)	0.8 days	5.2 days	0.32
Patients with a hospitalization in the 30 days following index event	29.6%	25.0%	0.64
Follow-up days (mean per patient)	343.1 days	307.1 days	0.11
Number of cardiac imaging procedures and cardiac catheterizations (mean per patient)	2.5	1.4	0.01
Number of cardiac rehabilitation unit admittances (mean per patient)	7.5	7.2	0.97
ICD implantation	36.4%	0%	< 0.0001

DME: durable medical equipment; ICD: implantable cardioverter-defibrillator; SNF: skilled nursing facility.
